# Quality and Reliability of YouTube Videos on the Periodontal Tunnel Technique and Their Coverage of Procedure-Specific Content: A Cross-Sectional Study

**DOI:** 10.7759/cureus.116932

**Published:** 2026-09-25

**Authors:** Sahin Altug, Huseyin Akilli, Zeliha Basak Cakir Erdil, Metin Calisir

**Affiliations:** 1 Department of Periodontology, Faculty of Dentistry, Adıyaman Üniversitesi, Adıyaman, TUR

**Keywords:** gingival recession, plastic periodontal surgery, social media, tunnel technique, video quality, youtube videos

## Abstract

Objective

This study aimed to evaluate the quality, reliability, and informational content of YouTube videos (Alphabet Inc., Mountain View, California, United States) related to the periodontal tunnel technique and to examine technique-specific surgical content coverage.

Materials and methods

This cross-sectional study included 22 YouTube videos that met the inclusion criteria. The videos were independently evaluated by three periodontists using the Global Quality Scale (GQS), Journal of the American Medical Association (JAMA) benchmark criteria, DISCERN, and a study-specific 16-item Tunnel Content Score developed with reference to the literature and expert consensus. Inter-rater reliability was assessed using the intraclass correlation coefficient (ICC) and Fleiss’ κ. Mean scores from the three reviewers were used in the main analyses. Associations between quality scores and video engagement metrics were assessed using Spearman correlation analysis, and differences according to uploader type were evaluated using the Kruskal-Wallis test.

Results

Inter-rater reliability was moderate for GQS and JAMA and excellent for DISCERN and the Tunnel Content Score. Median scores were 3.00 for GQS, 1.00 for JAMA, 38.00 for DISCERN, and 10.17 for the Tunnel Content Score. GQS was strongly correlated with DISCERN (ρ=0.737; *p*<0.001), while DISCERN was moderately correlated with the Tunnel Content Score (ρ=0.494; *p*=0.026). No significant associations were found between video engagement metrics and quality scores, and no significant differences were observed according to uploader type (all *p*>0.05). Tunnel preparation and papilla preservation were included in all videos, whereas postoperative care and complications were absent. Recession classification (9.1%) and indications (31.8%) were less frequently represented.

Conclusion

YouTube videos related to the periodontal tunnel technique showed variability in quality, reliability, and content coverage. Technical procedural steps were more prominently represented, whereas content related to clinical decision-making and postoperative patient management was more limited. Therefore, these videos may be considered supplementary materials supporting structured clinical education and scientific sources. Studies with larger samples may contribute to supporting these findings.

## Introduction

Gingival recession is a mucogingival condition characterized by exposure of the root surface as a result of apical displacement of the gingival margin relative to the cementoenamel junction [1]. Periodontal phenotype, tooth position within the alveolar arch, traumatic factors, periodontal diseases, and various local factors may play a role in its etiology. Gingival recession may be associated with esthetic concerns, dentin hypersensitivity, and root surface-related problems [2].

Various periodontal plastic surgical approaches are used in the management of gingival recession to achieve coverage of the exposed root surface and improve periodontal soft tissues, including coronally or laterally positioned flaps, free gingival grafts, subepithelial connective tissue grafts, and tunnel techniques [3]. Among these, the tunnel technique is a root coverage approach based on the preparation of a tunnel beneath the soft tissues while preserving papillary integrity, and several modifications of the technique have been described over time [4].

Detailed articles describing this technique, which involves several surgical steps such as tunnel preparation, graft placement and stabilization, coronal positioning of the tissues, and suturing, are available [5], as are online video resources. YouTube (Alphabet Inc., Mountain View, California, United States) is increasingly being used by dental students as an adjunctive source for learning clinical procedures. In a multicenter study of dental students, most respondents considered YouTube videos helpful for learning clinical procedures, although a substantial proportion remained uncertain about the evidence base of the available content [6]. Periodontal surgery-related YouTube videos, including those on free gingival grafting and gingival graft procedures, have also been evaluated as potential sources of patient and student information, highlighting the need to assess their quality, reliability, and content coverage [7,8]. Accordingly, YouTube may serve as an accessible supplementary resource for periodontal surgical education, provided that its content is critically evaluated alongside formal education and evidence-based sources

However, health-related content on YouTube does not undergo a standardized scientific evaluation process, which may warrant consideration of the informational content, reliability, and quality of these videos. Tools such as the Global Quality Scale (GQS), the Journal of the American Medical Association (JAMA) benchmark criteria, and DISCERN have been used in studies evaluating health-related YouTube videos [9-12]. Nevertheless, data jointly evaluating the overall quality, reliability, and technique-specific surgical content coverage of YouTube videos on the periodontal tunnel technique remain limited.

The aim of this study was to evaluate the quality, reliability, and informational content of YouTube videos related to the periodontal tunnel technique using the GQS, JAMA benchmark criteria, and DISCERN, and to examine technique-specific content coverage using a 16-item Tunnel Content Score instrument developed specifically for this study. In addition, relationships among these scores, their associations with video engagement characteristics, and differences according to uploader type were evaluated. The null hypothesis (H₀) was that there would be no significant relationships among quality, reliability, and technical content scores or between these scores and video engagement characteristics, and that the scores would not differ significantly according to uploader type.

## Materials and methods

In this cross-sectional study, publicly available YouTube videos on the periodontal tunnel technique were evaluated for quality, reliability, and technique-specific content using predefined assessment tools. As only publicly available content was analyzed without involving human participants or personal data, ethics committee approval was not required.

Search strategy

A structured video search was conducted through Google Search (Google LLC, Mountain View, California, United States) on August 20, 2026, to identify publicly available YouTube videos related to the periodontal tunnel technique. A newly created Google account associated with a previously unused email address was used for the search. The account had no prior search or viewing history, and browser history and cookies were cleared before the search to minimize personalization based on previous user activity.

Each search term was entered separately into Google Search, after which the “Videos” tab was selected. Using the search tools, the source was restricted to YouTube, the language was restricted to English, and the video duration was restricted to the predefined “Medium” category (4-20 minutes). The search scope was set to “Worldwide,” and the date range was restricted to January 1, 2006, through August 1, 2026. No manual sorting option was applied; the videos were screened in the default relevance-based order returned by Google Search.

The following search terms were used separately: “tunnel technique”, “tunneling technique”, “coronally advanced tunnel”, “coronally advanced tunnel technique”, “modified coronally advanced tunnel”, “The Tunneled Coronally Advanced Flap”, “Laterally closed tunnel technique”, and “Vestibular Incision Subperiosteal Tunnel Access Technique”. The approximate number of results displayed by Google Search for each search term was recorded, yielding a total of 167,504 displayed results before application of the advanced filters. After filtering, 26 potentially eligible videos were identified and constituted the screening pool. One video that was used for reviewer calibration and three duplicate records were excluded, resulting in 22 videos for the final analysis. When the same video was identified through more than one search term, it was considered a single record.

The videos included in the screening pool were subsequently reviewed and scored independently by three periodontists between August 20 and 25, 2026.

Inclusion and exclusion criteria

Videos related to the periodontal tunnel technique, presented in English, and with a duration of 4-20 minutes were included in the study. This duration range was selected before screening because it corresponds to the “Medium” category in Google Search’s built-in video-search filters, which classify videos as short (<4 minutes), medium (4-20 minutes), or long (≥20 minutes). Because the videos were identified through the Google Search “Videos” tab with the source restricted to YouTube, this platform-specific filter provided a repeatable and standardized screening criterion. Medium-length videos were considered more likely to provide a structured description or demonstration of the procedure than very short clips, while remaining distinct from longer lecture- or webinar-style content. This approach is consistent with previous analyses of dental and implant-related YouTube videos [13-15].

Videos were considered to be solely promotional when their primary purpose was to advertise a product, material, instrument, clinic, or service and they did not provide substantive information or a demonstrable procedural step related to the periodontal tunnel technique. Videos uploaded by commercial organizations were not excluded solely on the basis of the uploader category; they were retained when they contained relevant educational or procedural content. The promotional-content criterion was applied during the initial eligibility screening by three periodontologists (SA, HA, and ZBÇE). Any uncertainty or disagreement was resolved through consensus with MÇ.

After applying the eligibility criteria and excluding three duplicate records and one reviewer calibration video from the screening pool of 26 videos, 22 videos were included in the final analysis (Figure 1).

**Figure 1:**
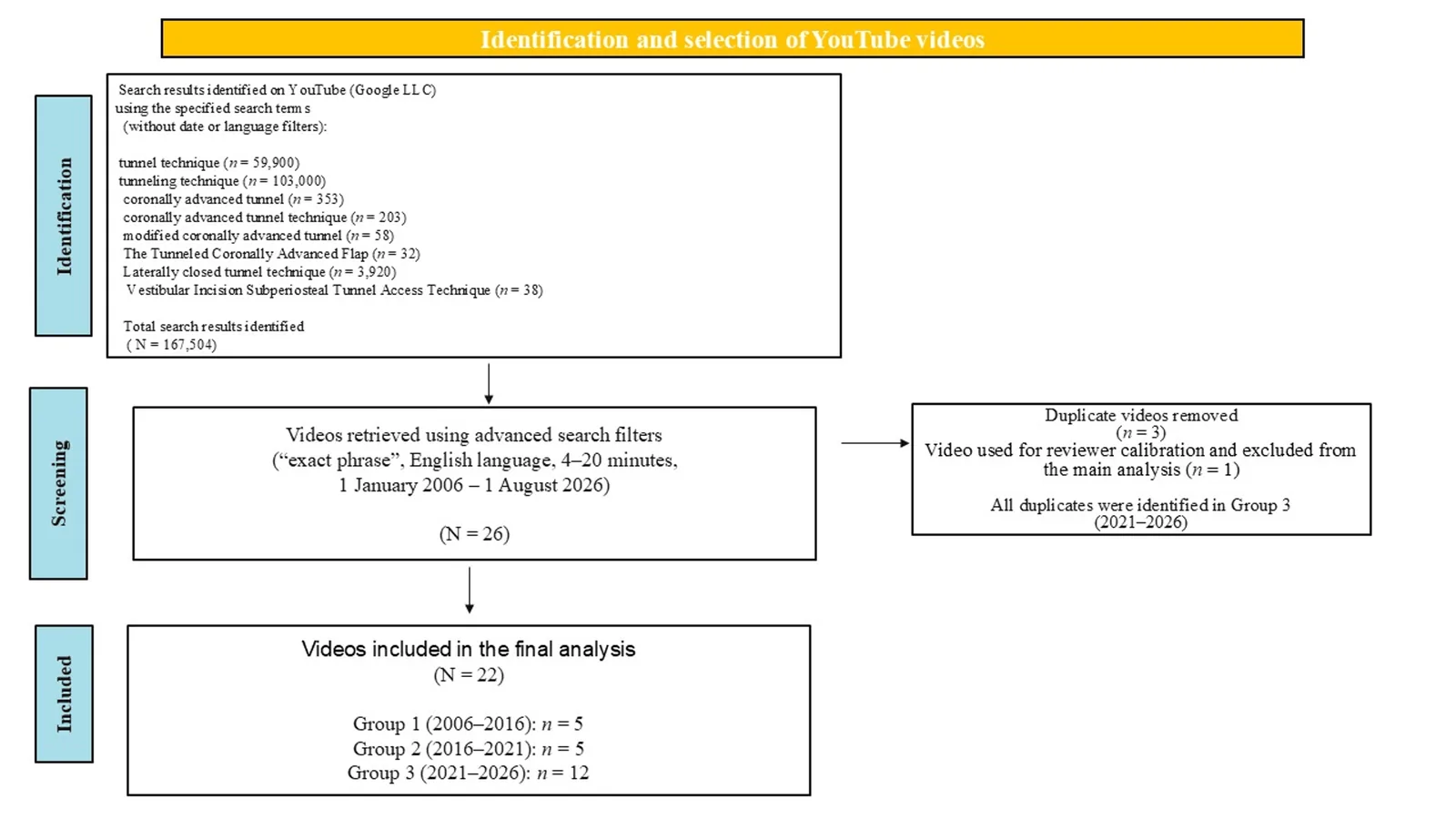
Flow diagram of the YouTube video selection process. The flow diagram summarizes the identification, screening, duplicate removal, calibration, and final inclusion of YouTube videos (Alphabet Inc., Mountain View, California, United States).

Data collection

For each included video, the URL, upload date, video duration, number of views, number of likes, and uploader type were recorded. YouTube video metadata were collected once on August 20, 2026, and were not subsequently updated. To account for differences in the length of time that videos had been available on the platform, the number of views per day (views/day) was calculated by dividing the total number of views as of August 20, 2026, by the number of days between the upload date and August 20, 2026.

Videos were classified according to the uploader categories recorded in the dataset: dentist/specialist, commercial organization/product account, educational platform/educational channel, and hospital/healthcare organization. The initial classification was performed by three periodontologists (SA, HA, and ZBÇE), and any uncertainty or disagreement was resolved through consensus with MÇ. The final analytical sample included 10 dentist/specialist, five commercial organization/product, four educational platform/educational channel, and three hospital/healthcare organization videos.

Assessment of video quality, reliability, and content

The videos included in the study were independently evaluated by three periodontists. The GQS [10], JAMA benchmark criteria [11], DISCERN [12], and a 16-item Tunnel Content Score specific to the tunnel technique were used for the evaluations. For the main analyses, the arithmetic mean of the scores assigned by the three reviewers was used for each video.

The GQS is a 5-point scale used to assess the overall quality of videos, with higher scores indicating higher overall quality [10]. The JAMA benchmark criteria consist of four criteria: authorship, attribution, disclosure, and currency. Each criterion is scored as 0 or 1, resulting in a total score ranging from 0 to 4 [11]. DISCERN was applied in its standard 16-item format. The instrument consists of 15 assessment questions and one overall quality rating item, with each item scored from 1 to 5 and a total score ranging from 16 to 80. The items were used to evaluate the reliability and quality of the health-related information presented in the videos, including the description of treatment options [12]. The complete item definitions and scoring structures for the GQS, JAMA benchmark criteria, DISCERN, and the study-specific Tunnel Content Score are provided in the Appendices (Appendix A-E).

A study-specific 16-item Tunnel Content Score (see Appendix E) was developed to assess the breadth of procedure-specific information presented in videos on the periodontal tunnel technique. The domains were selected with reference to relevant periodontal literature, including the systematic review and meta-analysis by Tavelli et al. [16], the technical review by Zuhr et al. [17], the clinical report by Pandit et al. [4], and the technical description by Marques et al. [5]. These publications describe the clinical indications, papilla preservation, tunnel preparation, graft-related procedures, tissue advancement, suturing, minimally invasive modifications, and clinical outcomes associated with tunnel procedures.

The literature-derived domains were reviewed and finalized through consensus among four periodontists. Before video assessment, operational definitions and coding rules were established for each item. The 16 domains comprised indication, gingival-recession classification, tunnel preparation, papilla preservation, root-surface preparation, root-surface conditioning, graft selection, graft harvesting, graft placement, graft stabilization, coronal positioning of the tissues, suturing, postoperative care, complications, clinical outcomes, and microsurgical approach. Each item was scored dichotomously as 0 (absent) or 1 (present), based solely on whether the relevant content was shown or described in the video. All items were equally weighted because no validated empirical basis was available for assigning differential weights; this did not imply that all domains were clinically equivalent in importance. Items such as root-surface conditioning, graft harvesting, and microsurgical magnification were considered relevant content domains but were not regarded as mandatory for every tunnel-technique procedure or modification. The total score ranged from 0 to 16.

The score assessed the presence or absence of predefined content domains and did not evaluate the accuracy, completeness, technical correctness, quality, or consistency of the information with current evidence. Formal content-validity indices and comprehensive psychometric validation were not performed.

Reviewer calibration

Before the main evaluation, three experienced periodontists independently assessed one calibration video using the predefined scoring criteria and the coding guide provided in Appendices A-E. The calibration video was used to standardize the application of the GQS, JAMA, DISCERN, and Tunnel Content Score criteria and was excluded from the final analysis. Following independent calibration, the reviewers compared their ratings and discussed any discrepancies with reference to the relevant scale instructions and the operational definitions of the study-specific items. Disagreements were reviewed and resolved by MÇ, an experienced senior periodontologist involved in the development of the study-specific assessment criteria. No formal minimum agreement threshold was prespecified before the main assessment. Reviewers could view the publicly available information displayed on the video pages; however, uploader identity, view count, likes, upload date, and other metadata were not used as scoring criteria. The Google-based video search and metadata collection were performed on August 20, 2026, whereas reviewer calibration and the independent assessment of the eligible videos were conducted between August 20 and August 25, 2026.

Statistical analysis

Before statistical analysis, the datasets were checked for completeness and consistency. Total JAMA, DISCERN, and Tunnel Content scores were recalculated from the item-level scores, and data entries were verified against the valid score ranges of the respective scales. Different labels used for uploader types were consolidated into standardized categories.

All statistical analyses were performed on the 22 videos comprising the main study sample. The video used for calibration was not included in the main analyses. Inter-rater reliability for GQS, total JAMA, total DISCERN, and Tunnel Content Score was assessed using intraclass correlation coefficients (ICCs) based on a two-way random-effects, absolute-agreement model. ICC(2,1) and ICC(2,k) were calculated for single measurements and the average measurements of the three reviewers, respectively. Inter-rater agreement for the four binary items of the JAMA criteria and the 16 binary items of the Tunnel Content Score was assessed using Fleiss’ kappa coefficient.

For the main analyses, the arithmetic mean of the scores assigned by the three reviewers was used for each video. Data distributions were assessed using the Shapiro-Wilk test. Exploratory associations among video quality, reliability, content, and engagement variables were examined using Spearman’s rank correlation coefficient. No primary correlation endpoint was prespecified, and no formal multiplicity adjustment was applied to these exploratory analyses. Two-sided nominal p-values and 95% confidence intervals (CIs) were calculated using Fisher’s z transformation and the Bonett-Wright standard error. The correlation findings were interpreted cautiously as hypothesis-generating, particularly given the small sample size.

All statistical tests were two-sided, and the level of statistical significance was set at p < 0.05. Analyses were performed using Python 3.12 (pingouin 0.6, statsmodels 0.15, scikit-posthocs, and SciPy 1.17; Python Software Foundation, Wilmington, Delaware, United States). ICC values were interpreted according to the classification of Koo and Li as <0.50, poor; 0.50-0.75, moderate; 0.75-0.90, good; and >0.90, excellent [18]. Kappa values were interpreted according to the Landis and Koch classification as ≤0.20, slight; 0.21-0.40, fair; 0.41-0.60, moderate; 0.61-0.80, substantial; and 0.81-1.00, almost perfect agreement [19].

## Results

Inter-rater reliability ranged from moderate to excellent across the total scores evaluated. The single-measure ICC (2,1) values were 0.68 (95% CI: 0.46-0.84) for GQS and 0.53 (95% CI: 0.28-0.74) for the total JAMA score, whereas the corresponding values for the total DISCERN score and Tunnel Content Score were 0.94 (95% CI: 0.86-0.98) and 0.99 (95% CI: 0.97-0.99), respectively. When the average measurements of the three reviewers were considered, the ICC(2,k) values were 0.87, 0.77, 0.98, and 1.00 for GQS, JAMA, DISCERN, and Tunnel Content Score, respectively. In the item-level analyses, Fleiss’ κ values for the JAMA items ranged from 0.45 to 0.72, with observed agreement ranging from 81.8% to 90.9%. For the Tunnel Content items, Fleiss’ κ values ranged from −0.02 to 1.00 for the 12 items for which κ could be calculated, while observed agreement across all 16 items ranged from 95.5% to 100.0%. Fleiss’ κ could not be calculated for TC3 (tunnel preparation), TC4 (papilla preservation), TC13 (postoperative care), and TC14 (complications) because there was no variability in the ratings (Table 1).

**Table 1:** Inter-rater reliability and agreement across assessment measures ICC(2,1) and ICC(2,k) denote single- and average-measure intraclass correlation coefficients, respectively, based on a two-way random-effects, absolute-agreement model. Fleiss’ κ values are presented as item-level ranges and do not represent overall κ estimates. For four Tunnel Content items, κ was not estimable because of invariant ratings; these items were excluded from the κ range but retained when determining the observed-agreement range. P values refer to ICC estimates. ICC, intraclass correlation coefficient; CI, confidence interval; κ, Fleiss’ kappa; GQS, Global Quality Score; JAMA, Journal of the American Medical Association benchmark criteria.

Measure	Reliability metric	Reliability estimate	95% CI	Observed agreement (%)	*P* value
GQS	ICC(2,1) / ICC(2,k)	0.68 / 0.87	0.46–0.84 / 0.72–0.94	—	<0.001
JAMA	ICC(2,1) / ICC(2,k)	0.53 / 0.77	0.28–0.74 / 0.53–0.90	—	<0.001
	Fleiss’ κ range (4 items)	0.45–0.72	—	81.8–90.9	—
DISCERN	ICC(2,1) / ICC(2,k)	0.94 / 0.98	0.86–0.98 / 0.95–0.99	—	<0.001
Tunnel Content Score	ICC(2,1) / ICC(2,k)	0.99 / 1.00	0.97–0.99 / 0.99–1.00	—	<0.001
	Fleiss’ κ range (12/16 estimable items)	−0.02–1.00	—	95.5–100.0	—

A total of 22 videos were included in the analysis. The Shapiro-Wilk test indicated that the evaluated variables, except for video duration, were not normally distributed. The median video duration was 516.00 seconds (Q1-Q3: 385.50-796.25), the median number of views was 6,050 (Q1-Q3: 1,580-14,250), and the median number of likes was 63.50 (Q1-Q3: 24.75-174.75). The median number of views per day was 3.24 (Q1-Q3: 0.90-9.50). Based on the mean scores of the three reviewers, the median GQS, JAMA, DISCERN, and Tunnel Content Score values were 3.00 (Q1-Q3: 2.75-3.33), 1.00 (Q1-Q3: 1.00-1.33), 38.00 (Q1-Q3: 36.33-38.92), and 10.17 (Q1-Q3: 10.00-11.00), respectively (Table 2). Of the videos, 45.5% were uploaded by dentists/specialists, followed by commercial organizations (22.7%), educational platforms (18.2%), and hospitals/healthcare organizations (13.6%) (Table 3).

**Table 2:** Descriptive characteristics of the analysed videos (N = 22) Views per day were calculated using 20 August 2026 as the census date. Normality was assessed using the Shapiro–Wilk test, and the corresponding p-values are reported. SD: standard deviation; Q1: first quartile; Q3: third quartile; GQS: Global Quality Score; JAMA: Journal of the American Medical Association benchmark criteria

Variable	Mean ± SD	Median (Q1–Q3)	Range	Shapiro–Wilk test, p
Duration (seconds)	601.09 ± 277.33	516.00 (385.50–796.25)	248.00–1,177.00	0.153
Views	16,236.59 ± 35,545.97	6,050.00 (1,580.00–14,250.00)	398.00–168,000.00	< 0.001
Likes	151.23 ± 195.63	63.50 (24.75–174.75)	0.00–696.00	< 0.001
Views per day	11.59 ± 21.66	3.24 (0.90–9.50)	0.24–95.35	< 0.001
GQS	2.98 ± 0.79	3.00 (2.75–3.33)	1.00–4.00	0.003
JAMA total	1.20 ± 0.44	1.00 (1.00–1.33)	1.00–3.00	< 0.001
DISCERN total	37.38 ± 6.06	38.00 (36.33–38.92)	26.00–57.67	< 0.001
Tunnel Content Score	9.89 ± 2.36	10.17 (10.00–11.00)	4.00–13.00	0.002

**Table 3:** Distribution of videos according to uploader categories

Category	Frequency (Percentage)
Dentist/Specialist	10 (45.5)
Commercial company	5 (22.7)
Educational platform	4 (18.2)
Hospital/Health institution	3 (13.6)

Spearman’s rank correlation analysis revealed a strong, positive correlation between GQS and the total DISCERN score (ρ = 0.737; 95% CI: 0.41-0.90; p < 0.001). A moderate, positive, and nominally significant association was also observed between the total DISCERN score and the Tunnel Content Score (ρ = 0.494; 95% CI: 0.06-0.77; p = 0.026). However, because the correlation analyses were exploratory and no multiplicity adjustment was applied, these findings should be interpreted cautiously, and the nominally significant association should be considered hypothesis-generating rather than confirmatory. Correlations among the other quality scores were not statistically significant. No statistically significant associations were observed between the number of views, number of likes, video duration, or views per day and GQS, JAMA, DISCERN, or Tunnel Content Score (all p > 0.05) (Table 4).

**Table 4:** Spearman correlations among quality scores and between engagement metrics and quality scores Correlations were assessed using Spearman’s rank correlation test. Reported p-values correspond to the Spearman correlation coefficients. ρ: Spearman correlation coefficient; CI: confidence interval; GQS: Global Quality Score; JAMA: Journal of the American Medical Association benchmark criteria

Variable 1	Variable 2	Spearman ρ	95% CI	p
GQS	JAMA total	0.218	−0.23 to 0.59	0.339
GQS	DISCERN total	0.737	0.41 to 0.90	< 0.001
GQS	Tunnel Content Score	0.398	−0.05 to 0.71	0.077
JAMA total	DISCERN total	0.037	−0.39 to 0.45	0.871
JAMA total	Tunnel Content Score	0.356	−0.09 to 0.68	0.116
DISCERN total	Tunnel Content Score	0.494	0.06 to 0.77	0.026
Views	GQS	0.016	−0.41 to 0.43	0.944
Views	JAMA total	−0.094	−0.50 to 0.34	0.681
Views	DISCERN total	−0.176	−0.56 to 0.27	0.442
Views	Tunnel Content Score	−0.421	−0.72 to 0.02	0.061
Likes	GQS	0.249	−0.20 to 0.61	0.276
Likes	JAMA total	0.166	−0.28 to 0.55	0.469
Likes	DISCERN total	−0.081	−0.49 to 0.35	0.723
Likes	Tunnel Content Score	0.026	−0.40 to 0.44	0.909
Video duration	GQS	−0.109	−0.51 to 0.33	0.633
Video duration	JAMA total	−0.151	−0.54 to 0.29	0.510
Video duration	DISCERN total	0.012	−0.41 to 0.43	0.957
Video duration	Tunnel Content Score	0.192	−0.25 to 0.57	0.402
Views per day	GQS	0.434	−0.01 to 0.73	0.053
Views per day	JAMA total	0.094	−0.34 to 0.50	0.681
Views per day	DISCERN total	0.045	−0.38 to 0.46	0.845
Views per day	Tunnel Content Score	−0.138	−0.53 to 0.30	0.546

No statistically significant differences were detected in GQS, JAMA, DISCERN, or Tunnel Content Score according to uploader type in this sample (Kruskal-Wallis test: H(3) = 6.50, p = 0.090; H(3) = 6.80, p = 0.079; H(3) = 6.32, p = 0.097; and H(3) = 4.80, p = 0.187, respectively). The corresponding epsilon-squared (ε²) effect sizes were 0.310, 0.324, 0.301, and 0.229, respectively (Table 5). Given the small and uneven subgroup sizes (n = 3-10), these nonsignificant results should not be interpreted as demonstrating equivalence between uploader groups.

**Table 5:** Comparison of quality scores according to uploader type Comparisons among uploader types were performed using the Kruskal–Wallis test. The Kruskal–Wallis H statistic (df = 3), corresponding p-values, and ε² effect sizes are reported. Q1: first quartile; Q3: third quartile; GQS: Global Quality Score; JAMA: Journal of the American Medical Association benchmark criteria

Uploader type	n	GQS, median (Q1-Q3)	JAMA total, median (Q1-Q3)	DISCERN total, median (Q1-Q3)	Tunnel Content Score, median (Q1-Q3)
Dentist / Specialist	10	3.00 (2.17–3.33)	1.00 (1.00–1.00)	38.00 (35.33–38.92)	10.50 (10.00–11.00)
Commercial company	5	3.00 (3.00–3.00)	1.00 (1.00–1.33)	36.33 (34.33–37.00)	8.00 (8.00–10.00)
Educational platform	4	3.67 (3.58–3.75)	1.50 (1.25–2.00)	38.83 (38.25–43.92)	11.17 (10.25–12.25)
Hospital/healthcare institution	3	3.00 (2.83–3.50)	1.00 (1.00–1.00)	38.67 (38.00–38.83)	12.00 (11.00–12.00)
Kruskal–Wallis H (df = 3)		6.50	6.80	6.32	4.80
p		0.090	0.079	0.097	0.187
ε² (epsilon-squared)		0.310	0.324	0.301	0.229

Regarding the distribution of the Tunnel Content Score items, tunnel preparation and papilla preservation were addressed in all videos (100%). Graft stabilization, coronal positioning, and suturing were included in 90.9% of the videos, while graft selection, graft placement, and microsurgery were included in 86.4%. In contrast, recession classification was addressed in only 9.1% of the videos, and information on indications was included in 31.8%. Information on postoperative care and complications was not provided in any of the videos (Figure 2).

**Figure 2:**
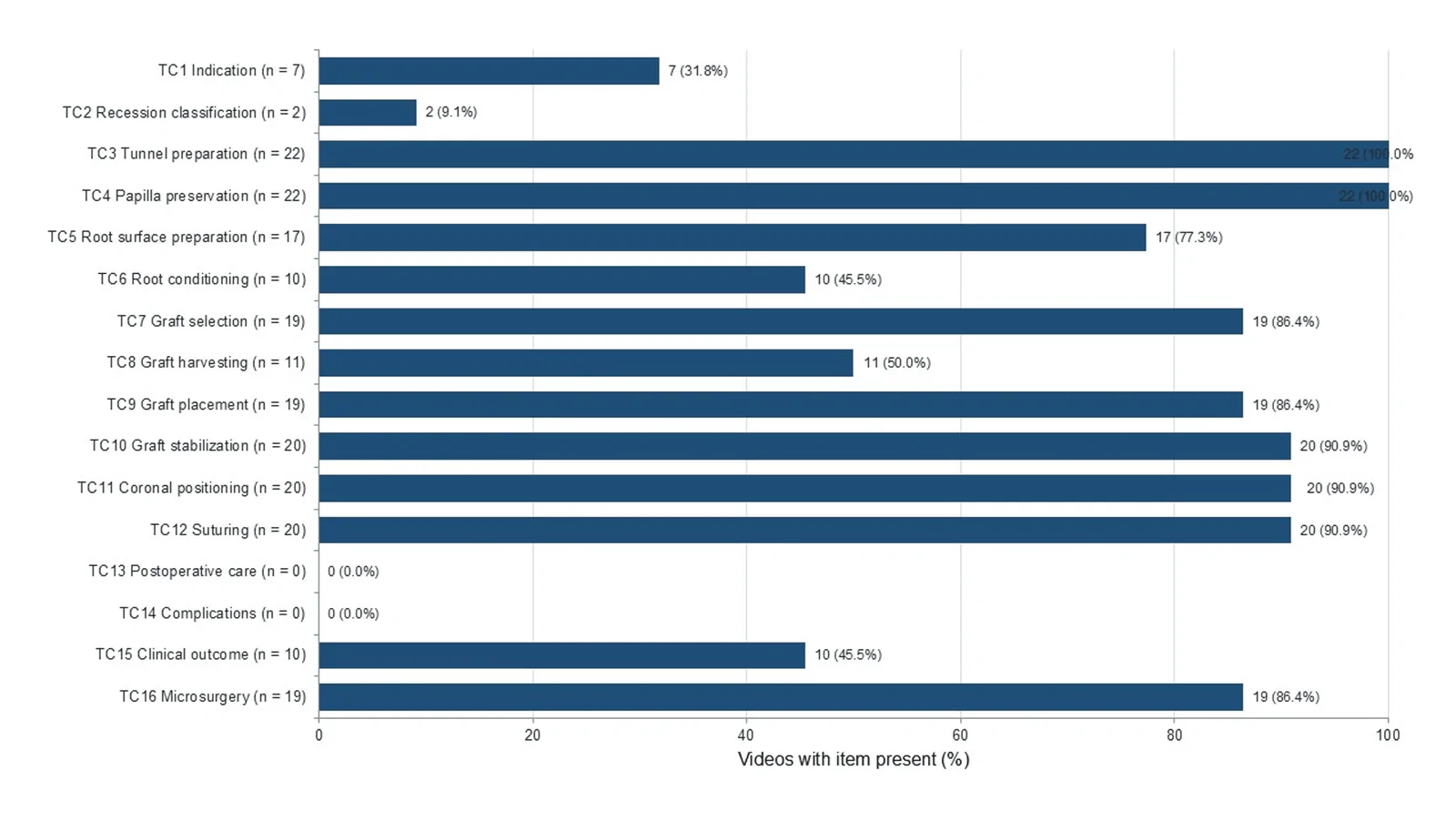
Frequency of Tunnel Content Score items across the analysed videos (N=22) The figure shows the number and percentage of videos in which each of the 16 Tunnel Content Score items was classified as present. For each item, the three reviewers’ binary ratings were averaged for each video; an item was classified as present when the mean score was greater than 0.50, corresponding to a positive rating by at least two of the three reviewers.

## Discussion

The periodontal tunnel technique is a detailed procedure involving multiple surgical steps. Therefore, this study evaluated YouTube videos not only in terms of overall quality and reliability but also according to their coverage of technique-specific surgical content, to provide a comprehensive assessment of their educational characteristics.

Exploratory analyses suggested a possible association between GQS and DISCERN and a moderate, nominally significant association between DISCERN and the Tunnel Content Score, whereas video engagement metrics were not significantly associated with the assessment scores, and no statistically significant differences were detected according to uploader type. The association between DISCERN and the Tunnel Content Score was nominally significant (ρ = 0.494; p = 0.026). Because the correlation analyses were exploratory and no multiplicity adjustment was applied, this finding should be interpreted cautiously as hypothesis-generating rather than confirmatory. These findings may indicate that the evaluated characteristics are not entirely independent, while video popularity and uploader source may not directly reflect the quality, reliability, or content measures examined.

The tools used to evaluate online health-related videos address different aspects of the content. In this study, the GQS was used to assess the overall quality and educational usefulness of the videos [10], the JAMA benchmark criteria to assess the reliability and transparency of information presentation [11,20], and DISCERN to assess the quality of health information [12]. However, these general assessment tools do not directly evaluate the extent to which surgical steps specific to the periodontal tunnel technique are represented in the videos. Therefore, a study-specific 16-item Tunnel Content Score tool covering the main components of the technique, from indications and recession classification to tunnel preparation, graft management, stabilization, suturing, and postoperative evaluation, was used [4,5,17,20]. The combined use of these tools allowed the overall quality and reliability of the videos and their technique-specific content coverage to be evaluated separately.

Inter-rater reliability varied across the assessment tools. The relatively lower reliability for GQS and JAMA may reflect their assessment structure and subjective components [10,11,20], whereas greater consistency was observed for DISCERN [12] and the Tunnel Content Score. The item-based structure of the Tunnel Content Score may have contributed to this consistency. However, variability in κ values despite high observed agreement could reflect imbalanced item distributions. Therefore, high reliability at the total-score level does not necessarily indicate equally strong agreement for each individual item.

Although the periodontal tunnel technique has been described with different modifications and has been widely addressed in the literature [5,17], only 22 videos were retained in our study after applying the predefined search and eligibility criteria, including the selected terminology, exact-phrase search, English-language restriction, duration criterion of 4-20 minutes, and specified publication-date range. Therefore, this sample represents the videos identified under our specific methodology. Considering the dynamic nature of YouTube, the potential for search results to change over time, and the influence of search terms and the platform’s ranking algorithms on the results, this number should not be considered an indicator of all relevant content available on the platform [21,22]. The use of different terminology or modified names for the same technique may also have resulted in some relevant videos falling outside the scope of the search.

In the evaluation of online health-related and surgical videos, “quality” is not a unidimensional concept [9]. A video may present a surgical procedure in a clear and easy-to-follow manner while not necessarily providing the same level of source transparency, reliability, or procedure-specific content coverage. The relatively independent pattern of the JAMA benchmark criteria compared with the other assessment tools may be related to their focus on aspects of information transparency, including authorship, attribution, currency, and disclosure [11]. These aspects may receive less emphasis in videos primarily focused on demonstrating a surgical procedure.

The partial overlap among the assessment measures may indicate that general information-quality assessments and procedure-specific content coverage are related but distinct dimensions. However, the presence of a greater number of procedure-specific elements does not establish that the surgical technique was demonstrated correctly, that the stated indications were clinically appropriate, or that the information was consistent with current periodontal evidence. Therefore, general quality and reliability measures should be interpreted separately from procedure-specific content coverage.

No statistically significant associations were detected between video engagement metrics and quality or content scores in this sample. Views and likes may be influenced by factors such as time since publication, channel reach, and user behavior, in addition to scientific and educational value [23,24]. Therefore, the absence of a statistically significant association in this study should not be interpreted as evidence that no relationship exists between popularity metrics and video quality or technical surgical content. Given the small sample size, these findings should be interpreted cautiously and should not be generalized.

Similarly, the absence of significant differences in quality and content scores according to uploader type suggests that uploader source alone may not reflect video quality. However, the small and uneven group sizes and the observed effect sizes limit definitive interpretation of this finding [25]. Further evaluation in larger and more balanced samples may therefore be appropriate.

Technique-specific analysis showed that procedural steps, including tunnel preparation, graft stabilization, coronal positioning, and suturing, were frequently represented, whereas indications, recession classification, postoperative care, and complications were less frequently addressed. This distribution may reflect the suitability of video-based content for demonstrating the visual and practical aspects of a procedure, while providing less information about case selection, risk assessment, clinical indications, and postoperative management [9,16,22]. Because the tunnel technique involves not only surgical execution but also assessment of recession characteristics, soft-tissue and graft management, and postoperative care [16,26], videos focusing primarily on technical steps may provide only part of the information required for clinical learning. Therefore, such videos may be most appropriately used together with evidence-based sources and structured clinical education [27].

Limitations

This study has several limitations. YouTube search results and engagement data may change over time and may be influenced by personalization and ranking algorithms, although the search was conducted using a newly created account without prior search or viewing history. Despite the standardized search strategy, videos using different terminology or modification names may have remained outside the search scope. The restriction to English-language videos within the predefined duration of 4-20 minutes and specified publication-date range, together with exact-phrase searching and selected terminology, may have excluded otherwise relevant content. Therefore, the findings may not be generalizable to videos in other languages, videos outside the specified duration range, or content excluded by the applied search filters. Uploader categories and promotional-content exclusions were based on predefined operational criteria, although some subjectivity in classification may remain. Reviewers were not blinded to publicly displayed video information; however, uploader type, views, likes, and other metadata were not used as scoring criteria.

The limited sample size and uneven uploader subgroups may limit the statistical power of subgroup comparisons and the generalizability of the findings. The multiple correlation and group-comparison analyses were exploratory, and not all analyses were prespecified or adjusted for multiplicity. The study-specific Tunnel Content Score has not undergone comprehensive psychometric validation and measures the presence or absence of predefined content elements rather than the accuracy, completeness, technical correctness, or consistency of the information with current evidence. All items were equally weighted, although their clinical importance may differ and some items may not be applicable to every tunnel-technique modification. Accordingly, the total score should be interpreted as a measure of content coverage rather than clinical accuracy or procedural quality. Finally, this study assessed only the content presented in the videos and could not determine its effects on viewers’ knowledge, clinical decision-making, or surgical performance.

## Conclusions

The YouTube videos evaluated in this study, which met the predefined search, language, duration, and publication-date criteria, showed variability in overall quality, reliability, and technique-specific content coverage. Technical procedural steps were more prominently represented, whereas components related to clinical decision-making, including indications, case assessment, complications, and postoperative management, received more limited coverage. These findings indicate that the educational value of online videos should not be assessed solely on the basis of their visual and technical presentation. Inclusion of content supporting clinical decision-making could enhance their educational value.

Given the limitations of the present assessment, these videos may be considered as supplementary visual resources when used alongside structured education and evidence-based scientific sources; however, their educational effectiveness and safety were not assessed in this study. Future studies should use larger video samples collected at different time points and evaluate learner-centered outcomes, including knowledge acquisition, procedural understanding, clinical decision-making, and skill performance. Formal validation of technique-specific content assessment tools, including the Tunnel Content Score, could strengthen the assessment of procedure-specific content but would not, by itself, establish educational effectiveness.

## Appendices

Appendix A 

**Table 6:** Coding rules for assessment tools JAMA and Tunnel Content Score items were coded as 0 (absent/no) or 1 (present/yes). GQS was scored from 1 to 5, and each DISCERN item was scored from 1 to 5. Total scores were calculated automatically from the corresponding item-level scores and were not entered manually. JAMA: Journal of the American Medical Association; GQS: Global Quality Score

Section/item	Operational definition or coding rule
JAMA benchmark criteria and Tunnel Content Score items	0 = absent/no; 1 = present/yes
GQS	One overall score ranging from 1 to 5
DISCERN items	Each item scored from 1 to 5
Total-score fields	Automatically calculated; not entered manually

Appendix B 

**Table 7:** JAMA benchmark criteria Each JAMA criterion was scored dichotomously as 0 (absent/no) or 1 (present/yes). The total JAMA score was calculated by summing the four item scores and ranged from 0 to 4. JAMA: Journal of the American Medical Association

Item	Operational definition	Coding
JAMA1. Authorship	Author, source, references, or professional credentials are identified.	0 = absent/no; 1 = present/yes
JAMA2. Attribution	Copyright information and information sources are clearly identified.	0 = absent/no; 1 = present/yes
JAMA3. Disclosure	Ownership, sponsorship, advertising, or conflicts of interest are disclosed.	0 = absent/no; 1 = present/yes
JAMA4. Currency	Publication date or date of updating is provided.	0 = absent/no; 1 = present/yes

Appendix C 

**Table 8:** Global Quality Scale Each video received one overall GQS score ranging from 1 to 5. Higher scores indicate better overall quality and educational usefulness. The GQS assesses overall presentation and usefulness; it does not specifically measure procedural accuracy. GQS: Global Quality Scale

Score	Operational definition
1	Poor quality; poor flow and very limited usefulness for patients
2	Generally poor quality; limited information and limited usefulness for patients
3	Moderate quality; some important information is presented adequately
4	Good quality; most relevant information is provided, with good flow and usefulness for patients
5	Excellent quality and flow; highly useful for patients

Appendix D 

**Table 9:** DISCERN instrument Each DISCERN item was scored from 1 to 5. The total DISCERN score was calculated by summing the 16 item scores and ranged from 16 to 80.

Item	Question/operational definition	Scoring
DISCERN1	Are the aims of the publication clear?	1–5
DISCERN2	Does the publication achieve its aims?	1–5
DISCERN3	Is the content relevant to the topic?	1–5
DISCERN4	Are the information sources identified?	1–5
DISCERN5	Is the date of production or updating stated?	1–5
DISCERN6	Is the information balanced and unbiased?	1–5
DISCERN7	Are additional sources of support and information provided?	1–5
DISCERN8	Are areas of uncertainty addressed?	1–5
DISCERN9	Is an explanation provided of how each treatment works?	1–5
DISCERN10	Are the benefits of each treatment explained?	1–5
DISCERN11	Are the risks of each treatment explained?	1–5
DISCERN12	Is it explained what might happen if treatment is not undertaken?	1–5
DISCERN13	Is the effect of treatment options on quality of life explained?	1–5
DISCERN14	Is it stated that more than one treatment option may be available?	1–5
DISCERN15	Does the information support shared decision-making?	1–5
DISCERN16	Overall quality of the publication as a source of information	1–5

Appendix E 

**Table 10:** Tunnel Content Score Each item was coded dichotomously according to whether the specified content was present in the video. The total score was calculated by summing the 16 item scores and ranged from 0 to 16. Items were equally weighted. The score measures the presence or absence of predefined content elements; it does not assess their accuracy, completeness, technical correctness, or consistency with current evidence. Some items may not be applicable to every modification of the tunnel technique. EDTA: ethylenediaminetetraacetic acid; SCTG: subepithelial connective tissue grafts; ADM: acellular dermal matrix

Item	Operational definition	Coding
TC1—Indication	The indication for the tunnel technique or case-selection criteria are stated.	0 = absent; 1 = present
TC2—Recession classification	A recognized classification system is explicitly identified, such as Miller Classes I–IV or Cairo RT1–RT3.	0 = absent; 1 = present
TC3—Tunnel preparation	Tunnel or pouch preparation is shown or described.	0 = absent; 1 = present
TC4—Papilla preservation	Preservation of papillary integrity is addressed.	0 = absent; 1 = present
TC5—Root-surface preparation	Root-surface preparation, including root planing or debridement, is addressed.	0 = absent; 1 = present
TC6—Root-surface conditioning	Root-surface conditioning, such as EDTA application, is addressed.	0 = absent; 1 = present
TC7—Graft selection	The graft type, such as SCTG, ADM, or collagen matrix, is specified.	0 = absent; 1 = present
TC8—Graft harvesting	Graft harvesting or the donor-site technique is shown or described.	0 = absent; 1 = present
TC9—Graft placement	Placement of the graft within the tunnel is shown or described.	0 = absent; 1 = present
TC10—Graft stabilization	Graft stabilization or suturing is addressed.	0 = absent; 1 = present
TC11—Coronal positioning	Coronal positioning or advancement of the flap/tissues is shown or described.	0 = absent; 1 = present
TC12—Suturing	The suturing technique, such as sling sutures, is shown or described.	0 = absent; 1 = present
TC13—Postoperative care	Postoperative instructions or care are described.	0 = absent; 1 = present
TC14—Complications	Potential complications are addressed.	0 = absent; 1 = present
TC15—Clinical outcome	Clinical outcomes or root-coverage results are presented.	0 = absent; 1 = present
TC16—Microsurgical approach	Use of microsurgical instruments or magnification, such as loupes or a microscope, is described.	0 = absent; 1 = present
